# Comparative proteome analyses of rhizomania resistant transgenic sugar beets based on RNA silencing mechanism

**DOI:** 10.1080/21645698.2021.1954467

**Published:** 2021-09-08

**Authors:** Sara Hejri, Azam Salimi, Mohammad Ali Malboobi, Foad Fatehi

**Affiliations:** a Department of Molecular Biotechnology, Institute of Agricultural Biotechnology, National Institute of Genetic Engineering and Biotechnology, Tehran, Iran; b Department of Plant Biology, Faculty of Biosciences, Kharazmi University, Tehran, Iran; c Department of Agriculture, Payame Noor University, Tehran, Iran

**Keywords:** Sugar beet, rhizomania, proteomics, mass spectrometry, substantial equivalence

## Abstract

Rhizomania is an economically important disease of sugar beet, which is caused by *Beet necrotic yellow vein virus* (BNYVV). As previously shown, RNA silencing mechanism effectively inhibit the viral propagation in transgenic sugar beet plants. To investigate possible proteomic changes induced by gene insertion and/or RNA silencing mechanism, the root protein profiles of wild type sugar beet genotype 9597, as a control, and transgenic events named 6018-T3:S6-44 (S6) and 219-T3:S3-13.2 (S3) were compared by two-dimensional gel electrophoresis. The accumulation levels of 25 and 24 proteins were differentially regulated in S3 and S6 plants, respectively. The accumulation of 15 spots were increased or decreased more than 2-fold. Additionally, 10 spots repressed or induced in both, while seven spots showed variable results in two events. All the differentially expressed spots were analyzed by MALDI-TOF-TOF mass spectrometry. The functional analysis of differentially accumulated proteins showed that most of them are related to the metabolism and defense/stress response. None of these recognized proteins were allergens or toxic proteins except for a spot identified as phenylcoumaran benzylic ether reductase, Pyrc5, which was decreased in the genetically modified S6 plant. These data are in favor of substantial equivalence of the transgenic plants in comparison to their related wild type cultivar since the proteomic profile of sugar beet root was not remarkably affected by gene transfer and activation RNA silencing mechanism.

## Introduction

Sugar beet, *Beta vulgaris*, is an herbaceous biennial plant from Amaranthaceae family that is cultivated in wide areas of the world to be used as food or feed.^1^ It provides a significant portion of the global demand for sugar because of high concentration of sucrose in its root. Also, it is used in bioethanol production. Therefore, it has a high commercial value.^2^

In recent decades, sugar beet has been exposed to an economically important disease designated as rhizomania. This devastating disease caused by *Beet necrotic yellow vein virus* (BNYVV) affects root quality and severely limits sugar yield. The most prominent symptoms of this viral infection are the development of numerous necrotic lateral roots around the main root (taproot) and the reduction of root size because of the restricted growth rate arising from the excessive proliferation of the lateral roots.^3–5^

The virus has four pathotypes, A, B, P and J, distributed in sugar beet fields around the world.^6^ BNYVV is transmitted via *Polymyxa betae* Keskin that produces resting spores which carry the virus and allow very long persistence in the soil even in the absence of the plant host^3–5^ to the extent that the application of chemical germicides for remediation of the virus or even rotation to non-host crops has not been effective.^3,4^

Since the discovery of *Rz* resistance genes, several classical plant breeding programs have been conducted to introduce resistance against BNYVV.^3,6^ However, despite the use of these genes, the virus can replicate at low levels.^5^ In recent years, several reports indicated the emergence of new pathotypes breaking the resistance mediated by *Rz 1* and *Rz2* in some areas.^6^ This necessitates exploring new methods and/or gene resources which control this pathogen. As a promising approach, genetic engineering by the use of RNA silencing induction^7^ or plantibody expression^8^ have been used to introduce transgenic sugar beet.

RNA silencing is a mechanism involving natural plant defense against viruses, gene expression regulation, and genome protection from transposons.^9,10^ In the latest progress in RNA silencing application, gene cassettes expressing RNA with hairpin RNA (hpRNA) structures are transferred into the target crops.^11^ It is shown that hpRNA is cleaved into small pieces called short interfering RNAs (siRNAs) by a dicer, an endoribonuclease RNase. siRNA connects to RNA-induced silencing complex (RISC), while a sense strand is disintegrated. RISC complex cleaves the RNA targets carrying the complementary sequence such as exogenous ones like viruses or even endogenous transcripts.^9,11,12^

Yet, a major public concern about cultivation of genetically modified (GM) plants and products thereof is the potential unintended effects of exogenous gene insertion and expression. Such possible effects necessitate the verification of “substantial equivalence” to the parental (wild type) plant. This include proteomics that are a set of techniques for identification and comparison of proteins affected by gene transfer leading to understanding possible alterations in metabolic reaction and molecular interactions in cellular pathways as well as probable accumulation of allergens or toxins.^13–17^

Sugar beet seeds used in this study have been genetically modified to induce resistance against rhizomania by triggering RNA silencing mechanism through the expression of intron-hairpin RNA (ihpRNA) structures. The inserted cassettes carry the 5ʹ-untranslated region (5ʹ-UTR) sequence of RNA2 with or without the gene sequence encoding P21 protein, named S3 and S6, respectively.

In this study, proteomes of two selected transgenic events, S3 and S6, were compared to their wild counterpart. Proteins were separated using two-dimensional gel electrophoresis (2DE) and differentially accumulated proteins were identified by mass spectrometry. Moreover, possible relations of the detected protein profile alterations with the gene transfer and the activation of RNA-silencing mechanism were considered.

## Materials and methods

### Plant cultivation and harvesting

In this study, we used sugar beets with the same genetic backgrounds including wild type variety, 9597, susceptible to BNYVV, as a control (provided by Sugar Beet Seed Research and Production Institute, Karaj, I.R. Iran), and two selected transgenic events, 219-T3:S3-13.2 (S3) and 6018-T3:S6-44 (S6) which were transformed by two different constructs, IHP‐P and IHP‐U, respectively. Both constructions IHP‐P and IHP‐U contained two copies of 5ʹ-untranslated region (5ʹ-UTR) sequence of RNA2 with or without the sequence encoding P21 protein, respectively, which were orientated in the sense and antisense with an intron expressing hpRNA in the middle of the construct.^7^ Homozygous sugar beet seeds from the third generation were planted in phytotron with a 16/8hs light/dark cycle at 25/20°C day/night temperature and a relative humidity of 60% in small pots containing equal amounts of autoclaved garden soil and sand. After 8 weeks, each plant was transferred into 1-L pot and was placed in a growth room with a 16/8hs light/dark photoperiod at 25–30°C temperature. All plants were harvested after 12 weeks; and were immediately and quickly washed to remove the soil. Roots were separated and frozen in liquid nitrogen prior to being stored at −70°C.

### DNA extraction and transgene amplification

Based on mini-Dellaporta method, genomic DNA was extracted from transgenic sugar beets^18^ and the present of transgenes was proved by PCR with transgenes-specific primers CaMV 35S-F (5´-CCACGTCTTCAAAGCAAGTGG-3´) and CaMV 35S-R [5´-TCCTCTCCAAATGAAATGAACTTC-3´). PCR reaction was performed by the first cycle at 94°C for 10 min, followed by 40 cycles at 94°C for 1 min, 60°C for 1 min, 72°C for 1 min and by the last cycle at 72°C for 10 min. The PCR products were separated by electrophoresis.

### Protein extraction and quantification

Root tissues of three biological replicates for each plant were grounded in liquid nitrogen using a mortar and pestle to make fine powder. Protein extraction was performed according to,^19^with few modifications. Root tissue was homogenized into 10% (w/v) trichloroacetic acid (TCA) in acetone containing 1 mM PMSF and 0.07% (v/v) 2-mercaptoethanol and incubated at −20°C overnight. Proteins were precipitated by centrifugation at 12,000rpm for 20 min at 4°C. Then, the pellet was washed three times with cold acetone containing 1 mM PMSF and 0.07% (v/v) 2-mercaptoethanol while after each washing step it was centrifuged at 12,000rpm for 2 min at 4°C. The pellet was finally air-dried at room temperature. The pellet was then dissolved in lysis buffer consisting of 35 mM Tris-HCl, pH 7, 7 M urea, 2 M thiourea, 1% (w/v) DTT, 4% (w/v) CHAPS and 1% (v/v) ampholyte pH 3.5–10 at room temperature for 1 h before centrifugation at 12,000 rpm for 15 min at 4°C. The supernatant was stored at −70°C and total protein content was quantified by Bradford method.^20^

### Two-dimensional Gel electrophoresis and spot selection

Isoelectric focusing (IEF) separation was carried out with IPG strips [linear, pH 4–7, 17 cm, Bio-rad, Hercules, CA, USA) according to.^21^ Briefly, IPG strips were rehydrated in 320 µl of rehydration solution (8 M urea, 20 mM DTT, 2% (w/v] CHAPS, 2% (v/v) IPG buffers (pH 3–10) and 0.002% Bromophenol blue) containing 120 µg proteins slot on a reswelling tray at room temperature for 12–16hs. IEF was performed at 20°C with Multiphor II (Amersham Pharmacia Biotech, UK) under the following conditions: 150Vh at 0–300 V, 300Vh at 300–500 V, 2000Vh at 500–3500 V, and lastly, 39,500Vh at 3500 V. After the first dimension, strips were incubated in equilibration buffer (50 mM Tris–HCl, pH 8.8, 6 M urea, 2% (w/v) SDS, 30% (w/v) glycerol, 1% (w/v) DTT and bromophenol blue) for 15 min.

For performing SDS-polyacrylamide gel electrophoresis (SDS-PAGE), IPG strips were placed on top of 12.5% gels in the Protean II Xi Cell electrophoresis system (Bio-Rad, Hercules, CA, USA). Proteins were stained with silver nitrate^22^ and gels were scanned using a GS800 Calibrated Imaging Densitometer (Bio-Rad, Hercules, CA, USA) in transmissive mode. Images were analyzed with the Melanie (ImageMaster) software version 6.0 (GE Healthcare, Amersham Biosciences, Uppsala, Sweden). Those spots that were present in all replicate gels were selected for further analysis. At least 2-fold alteration in signal intensity of spots among plants was taken as a threshold.

### In Gel Digestion and protein identiﬁcation

MassPREP automated digester station (PerkinElmer, Waltham, MA, USA) was used for in-gel digestion. After gel destaining using a solution of 15 mM potassium ferricyanide and 50 mM sodium thiosulfate 5 hydrate, protein reduction and alkylation were performed using 10 mM dithiotreitol (DTT) and 55 mM iodoacetamide (IAA), respectively, followed by a tryptic digestion in 50 mM ammonium bicarbonate, pH 8. Using a solution of 2% acetonitrile and 1% formic acid, peptides were taken out and then lyophilized.

Lyophilized peptides were dissolved in a solution of 0.1% TFA (trifluoroacetic acid) and 10% acetonitrile. 5 mg/mL of α-cyano-4-hydroxycinnamic acid (CHCA) as a MALDI matrix was prepared in 50% acetonitrile, 6 mM ammonium phosphate monobasic, and 0.1% trifluoroacetic acid. Then, it was mixed with the peptide at 1:1 ratio (v/v).

An AB Sciex 5800 TOF/TOF System and MALDI/TOF/TOF (Framingham, MA, USA) with a 349 nm Nd: YLF OptiBeam On-Axis laser were used for getting mass spectrometry data. The laser pulse rate was 400 Hz. Reflectron positive mode was applied while it was internally calibrated at 10 ppm mass tolerance and externally at 50 ppm. Each mass spectrum was collected as a sum of 500 shots.

### Protein characterization and classification

Using Mascot search engine (http://www.matrixscience.com), proteins were identified against Swiss-Prot with the following criteria: trypsin as enzyme, viridiplantae as taxonomy, carbamidomethyl (C) as fixed modification, oxidation (M) as variable modification and peptide tolerance (50ppm).^23^ Protein properties, locations, and functions were found at UniProt data base (https://www.uniprot.org/). The possible functions and roles of the annotated protein spots were looked for in Gene Ontology (http://geneontology.org/).

## Results

### Identification of differentially expressed proteins

**Figure 1.**
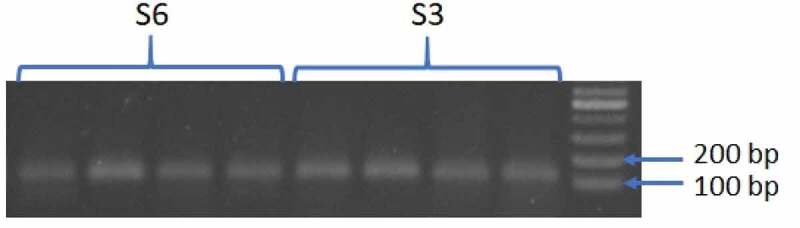
Reconfirmation of the presence of transgene in S3 and S6 events T3 off springs. Genomic DNA extractions were amplified by a pair of primers designed based on 35S-CaMV promoter sequence producing a 152 bp band in PCR.

**Figure 2.**
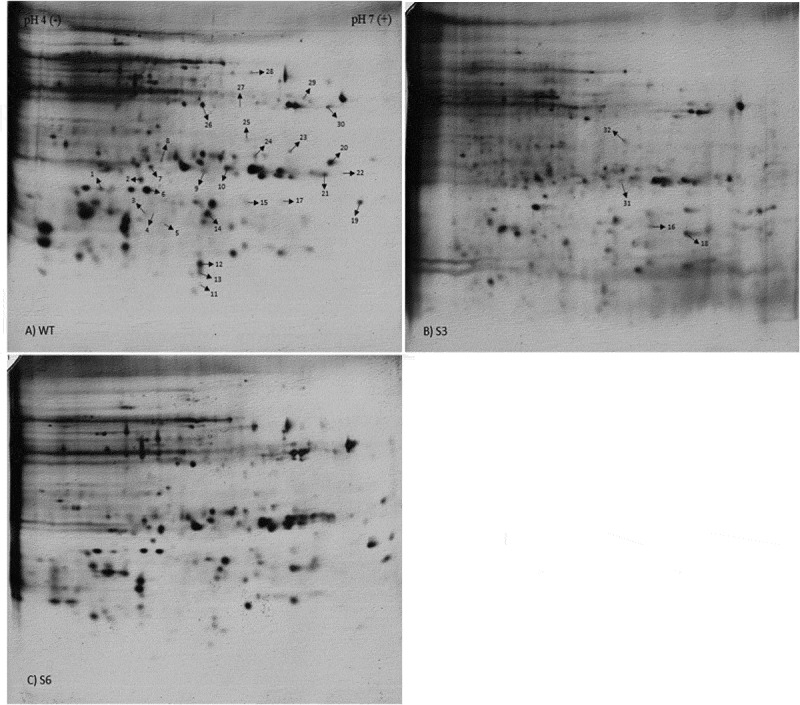
Representative 2-DE gel images of roots protein profiles. The first dimension was performed with 17 cm IPG strips (pH 4–7) and the second dimension with 12.5% SDS-polyacrilamide gels. Spots were visualized by silver staining of wild type (a), S3 (b), and S6 (c) transgenic plants. Proteins with differential accumulation levels are marked by arrows and their properties are listed in .Table 1

**Figure 3.**
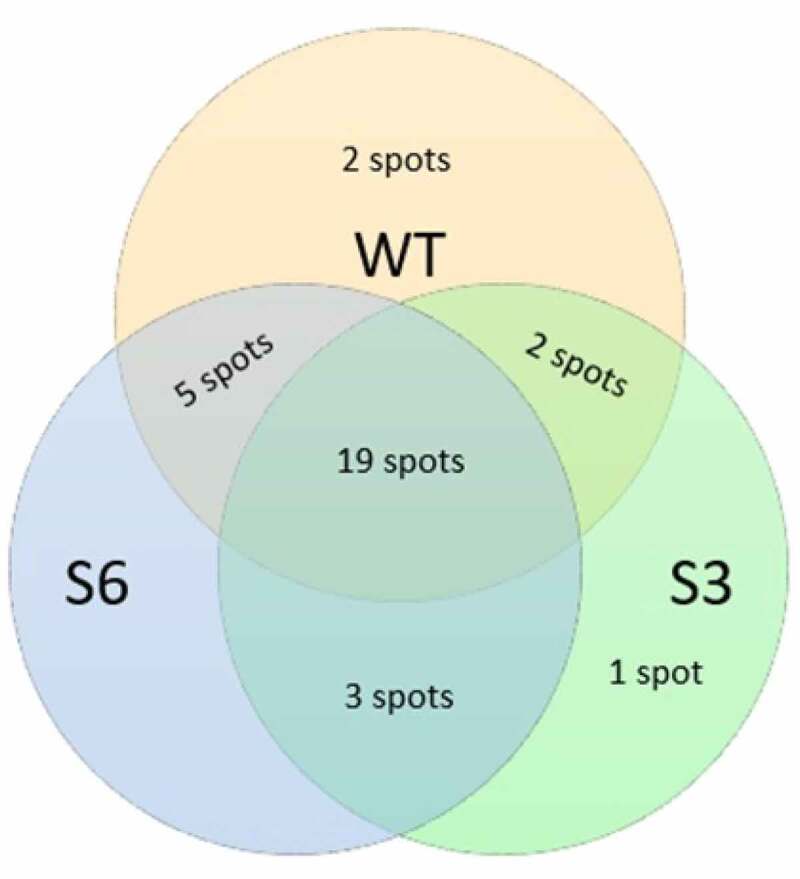
The number of differentially accumulated proteins in wild type, S3 and S6 transgenic plants.

In this study, two homozygous sugar beet events, produced through self-pollination to the third generation, were planted in the growth room. After reconfirming the presence of transgene by PCR in the selected events (Fig. 1), for finding the effect of transgene expression and subsequent RNA silencing activation against CP21 on protein profiles, we extracted root proteins of three biological replicates for each plant and then separated them using 2-DE technique. We first used IPG strips with a pH range of 3–10 to study the distribution of root proteins (data not shown). Since almost all proteins were in the range of 4 to 7, IPG strips with a pH rang of 4–7 were eventually chosen for the comparison of protein profiles among wild and transgenic plants in the first-dimension electrophoresis in order to have a higher resolution. After gels staining with silver nitrate and scanning, the images of gels were analyzed for identification of differentially expressed protein spots. Only the protein spots that revealed reproducible changes were used for further analysis. Out of 321 consistently detected spots in the gels, 32 proteins were found with at least 2-fold changes in their accumulation levels (Fig. 2). Specifically, nine (4, 5, 8, 13, 15, 19, 21, 24, and 30) and three (7, 25, and 29) spots were up-regulated in S3 and S6, respectively, while four other spots (6, 17, 22, and 28) were highly expressed in both transgenic events. Also, nine protein spots were less expressed in S6 (2, 5, 8, 9, 10, 12, 19, 26, and 30) and one spot was down-regulated in both transgenic plants (spot 14). Moreover, two spots were absent in both transgenic varieties compared to the non-transgenic parental plant (spots 3 and 20), whereas five (1, 12, 23, 26, and 27) and two (4 and 11) spots were not detected in S3 and S6, respectively. Additionally, in comparison with the wild cultivar, three proteins were only present in both transgenic events (spots 16, 18, and 32); however, one protein was specifically found in S3 (spot 31). In total, 10 spots showed presence or absence which is about 3% of all examined proteins. Nineteen spots were different in quantity (up-regulated or down-regulated) accounting for around 6% of the detected protein spots. Spots 4, 12, 26 were repressed in one transgenic event but showed expression variations in the other one. In total, 25 and 24 root proteins were affected in S3 and S6 transgenic plants, respectively (Fig. 3). Therefore, it is plausible that the proteome profiles of the transgenic events were not remarkably affected by gene insertion and activation of RNA silencing mechanism showing less than 8% changes.

Differentially expressed spots were proceeded to MALDI-TOF/TOF MS to reveal possible identities. Mascot program was used to search Swiss-Prot database looking for high score and sequence coverage criteria. Nearly 70% of these proteins had calculated pIs in the acidic and neutral pH range and 75% of them were distributed in the range of 10,000–50,000 Da. Protein properties including sample number, protein name, protein accession no., mascot score, protein sequence coverage%, monoisotopic mass, calculated pI, biological function, molecular function, subcellular locations, and protein status in the examined plants are presented in Table 1.

**Table 1.** Protein properties of differentially expressed root proteins

Spot No.	Annotations	UniProt Accession No. (name)	Score	sequence coverage%	Monoisotopic mass (M_r_) (Da)	Calculated pI	Molecular function	Biological role	Location	Accumulation status
1	1-aminocyclopropane-1-carboxylate synthase (Fragment)	Q01912 (1A1C_VI2GRR)	30	22	41,964	5.42	ACC synthase and pyridoxal phosphate binding activity	Stress response, Ethylene biosynthesis, Fruit ripening	Cytoplasm (Cytosol)	S3 *
2	Phospholipase D delta	Q9C5Y0 (PLDD1_ARATH)	43	22	99,595	6.71	Calcium binding and phospholipase activity	Stress response, Lipid metabolism	Plasma membrane	S6 ↓
3	Putative cytochrome c oxidase subunit II PS17 (Fragments)	P84733 (PS17_PINST)	30	100	1707	9.62	Electron transfer activity	Electron transfer in respiration process	Mitochondrion	S3 *, S6 *
4	Tyrosine–tRNA ligase 1, cytoplasmic	Q8S9J2 (SYYC1_ARATH)	39	23	44,439	6.31	ATP binding and tyrosine–tRNA ligase activity	Protein biosynthesis	Cytoplasm (Cytosol)	S6 *, S3 ↑
5	Protein PPLZ12	P16148 (PLZ12_LUPPO)	37	23	20,611	5.20	ND	ND	ND	S3 ↑, S6 ↓
6	Proteasome subunit beta type-2	Q9LST6 (PSB2_ORYSJ)	42	22	23,634	5.42	endopeptidase activity	Protein degradation	Nucleus, Cytoplasm	S3 ↑, S6 ↑
7	CST complex subunit STN1	Q9LMK5 (STN1_ARATH)	41	35	17,872	9.71	DNA binding activity	Telomer protection from degradation	Nucleus	S6 ↑
8	Vacuolar protein sorting-associated protein 22 homolog 1	Q5M759 (VP221_ARATH)	40	26	28,634	9.00	Endosomal sorting complex structural and protein transporter activity	Protein transport	Endosome	S3 ↑, S6 ↓
9	Phenylcoumaran benzylic ether reductase, Pyrc5	O81355 (PYRC5_PYRCO)	27	28	33,802	6.0	Phenylcoumaran benzylic ethers reductase activity	Lignan biosynthesis, Plant defense, Protection against oxidative damage	ND	S6 ↓
10	Glutaredoxin-C3	Q6K609 (GRXC3_ORYSJ)	52	58	14,372	6.89	Electron transfer and protein disulfide oxidoreductase activity	Electron transfer and reduce low molecular weight disulfides and proteins	Nucleus, Cytoplasm	S6 ↓
11	Putative aluminum-activated malate transporter 11	Q3E9Z9 (ALMTB_ARATH)	35	30	17,149	9.55	Ion channel	Ion transport	Plasma membrane	S6 *
12	Cytochrome P450 714D1	Q5KQH7 (C14D1_ORYSJ)	37	18	63,775	9.00	Heme binding and monooxygenase activity	Gibberellin catabolism	Endoplasmic reticulum membrane	S3 *, S6 ↓
13	Replication factor C subunit 4	Q7XRX1 (RFC4_ORYSJ)	32	31	37,178	6.54	ATP binding, DNA binding and ATPase activity	DNA replication and repair	Nucleus	S3 ↑
14	Probable serine/threonine-protein kinase PBL9	Q06548 (PBL9_ARATH)	44	14	45,776	9.60	ATP binding and protein kinase activity	Defense response	Plasma membrane	S3 ↓, S6 ↓
15	Cysteine proteinase inhibitor A	Q10992 (CYTA_HELAN)	50	64	9357	5.81	Cysteine type endopeptidase inhibitor activity	Inhibitor of papain and ficin (proteases)	ND	S3 ↑
16	Adenine nucleotide transporter BT1, chloroplastic/amyloplastic/mitochondrial	P29518 (BT1_MAIZE)	36	16	47,054	8.51	ATP transmembrane transporter activity	ADP transport, Purine nucleotide-sugar transmembrane transport	Mitochondrion inner membrane, Chloroplast inner membrane, Amyloplast inner membrane	S3 +, S6 +
17	NRR repressor homolog 1	Q6L589 (NRH1_ORYSJ)	43	35	19,350	4.72	Protein binding and inhibitor activity	Defense response	Nucleus	S3 ↑, S6 ↑
18	Aspartate aminotransferase P2, mitochondrial (Fragment)	P26563 (AATM_LUPAN)	50	19	50,056	7.64	Pyridoxal phosphate binding and aminotransferase activity	Carbon and energy metabolism, Nitrogen metabolism	Mitochondrion matrix	S3 +, S6 +
19	Monothiol glutaredoxin-S2	Q8L8Z8 (GRXS2_ARATH)	41	47	11,260	6.06	Electron transfer and metal ion binding and oxidoreductase activity	iron-sulfur protein assembly machinery, Electron transfer and reduce GSH-thiol disulfides	Cytoplasm	S3 ↑, S6 ↑
20	Probable trehalose-phosphate phosphatase 2	Q9FWQ2 (TPP2_ORYSJ)	52	32	42,779	5.84	Trehalose phosphatase activity	Trehalose biosynthesis,		
protect cellular entirety against abiotic stresses	Cytoplasm (Cytosol)	S3 *, S6 *								
21	Cysteine protease inhibitor 10 (Fragment)	O24383 (CPI10_SOLTU)	42	16	21,234	7.57	Cysteine type endopeptidase inhibitor activity	Protease inhibitor, Pathogen defense	Vacuole	S3 ↑
22	Ribonuclease J	Q84W56 (RNJ_ARATH)	40	11	101,061	8.42	Metal ion and mRNA binding and RNase activity	rRNA processing	Chloroplast	S3 ↑, S6 ↑
23	Probable pyridoxal 5ʹ-phosphate synthase subunit PDX2	Q8LAD0 (PDX2_ARATH)	39	36	27,535					
	5.18	Glutaminase and pyridoxal phosphate synthase activity	Vitamin B6 biosynthesis, Resistance to singlet oxygen-producing processes	Cytoplasm	S3 *					
24	Putative pentatricopeptide repeat-containing protein At1g12700, mitochondrial	P0C7Q7 (PPR38_ARATH)	41	28	68,559	7.88	ND	Mitochondrial mRNA modification	Mitochondrion	S3 ↑
25	Soluble inorganic pyrophosphatase	O23979 (IPYR_HORVV)	46	41	24,148	5.85	Magnesium ion binding and inorganic diphosphatase activity	Phosphate-containing compound metabolism	Cytoplasm	S6 ↑
26	Probable fructokinase-1	Q9SID0 (SCRK1_ARATH)	71	40	35,424	5.31	ATP binding and fructokinase activity	Carbohydrate metabolism	Plasma Membrane, Peroxisome, Cytosol	S3 *, S6 ↓
27	Eukaryotic translation initiation factor 3 subunit B	Q9C5Z1 (EIF3B_ARATH)	47	20	81,995	5.12	Translation initiation factor binding and activity	Protein biosynthesis	Cytoplasm	S3 *
28	Putative zinc finger A20 and AN1 domain-containing stress-associated protein 8	Q3EA33 (SAP8_ARATH)	44	60	14,198	8.74	DNA binding and zinc ion binding activity	Stress response	ND	S3 ↑, S6 ↑
29	Hypersensitive-induced response protein 4	Q9FHM7 (HIR4_ARATH)	41	36	32,586	5.28	ND	Defense against pathogen	Plasma membrane	S6 ↑
30	26.7 kDa heat shock protein, chloroplastic	Q10P60 (HS26P_ORYSJ)	43	42	26,705	6.78	ND	Stress response	Chloroplast	S3 ↑, S6 ↓
31	Lectin		40	33	23,819	5.62	Binding activity	Defense against pathogens and herbivorous insects	Plasma membrane	S3 +
32	Antiviral protein S	P23339 (RIPS_PHYAM)	52	51	29,410	8.86	rRNA N-glycosylase activity	Antiviral defense	ND	S3 +, S6 +

ND, no data.

1.The status of protein accumulation levels for S3 and S6 transgenic plants were compared with the wild type (WT). * absent, + present, ↑ up-regulated and ↓ down-regulated proteins.

### Functional classification of differentially expressed proteins

**Figure 4.**
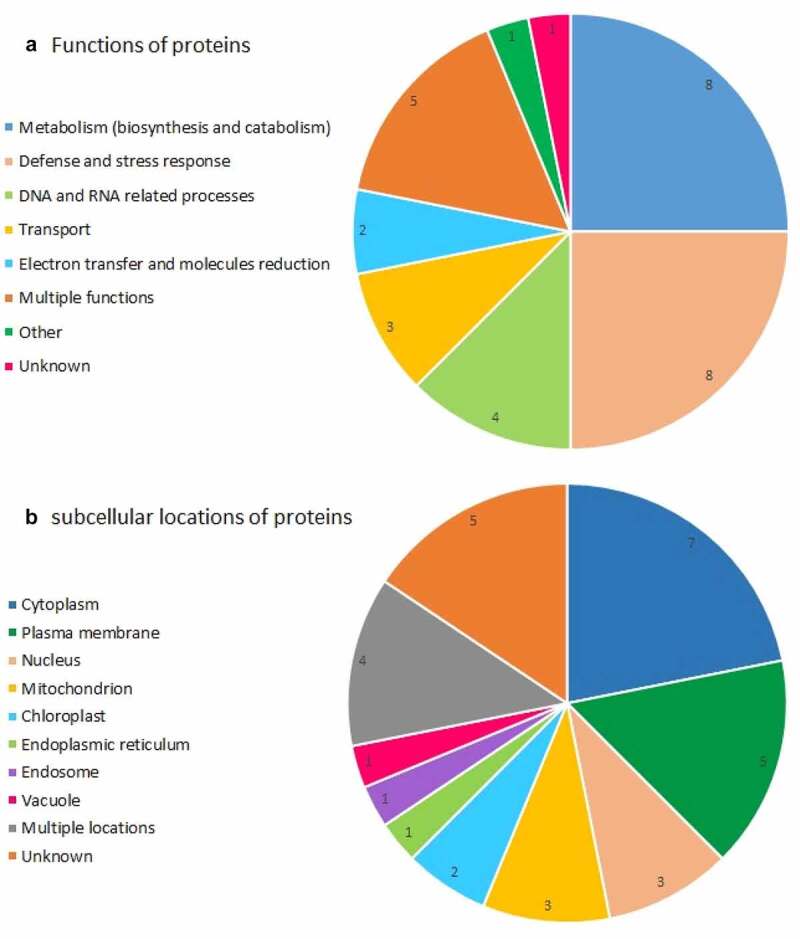
Functional (a) and subcellular (b) categories of the differentially accumulated protein numbers.

As shown in Fig. 4a, the differentially regulated proteins were classified into eight different groups based on their biological functions. Most of the proteins were related to the metabolism that were either involved in biosynthesis or in catabolism and energy processes (8 spots) or defense/stress responses (8 spots) followed by DNA and RNA processes (4 spots), transport (3 spots), and electron transfer (2 spots). Additionally, one spot was cysteine proteinase (papain and ficin) inhibitor. Some of them had multiple roles in both metabolism and defense response (5 spots).

### Subcellular localization of differentially expressed proteins

The identified proteins were predicted to be located in various compartments of the cell (Fig. 4b). The cytoplasm contained seven proteins out of 32 proteins (21.9%), including monothiol glutaredoxin-S2, probable pyridoxal 5ʹ-phosphate synthase subunit PDX2, soluble inorganic pyrophosphatase, translation initiation factor 3 subunit B, 1-aminocyclopropane-1-carboxylate synthase, Tyrosine–tRNA ligase 1, and trehalose-phosphate phosphatase 2, of which the last three proteins were only in the cytosol. Five proteins, being accounted for 15.7% of the spots, were in the plasma membrane, including phospholipase D delta, putative aluminum-activated malate transporter 11, probable serine/threonine-protein kinase PBL9, hypersensitive-induced response protein 4, and lectin. The mitochondria, and nucleus each comprised three spots, and two spots were placed in the chloroplast. The endosome, endoplasmic reticulum, and vacuole together contained three spots. The remaining proteins exhibited multiple subcellular locations (4 spots). For example, adenine nucleotide transporter BT1 exists in both mitochondrion and chloroplast. Also, there was no information about the subcellular status of 5 spots.

## Discussion

Sugar beet is one of the main industrial and agricultural crops in the world. With the advance of genetic engineering, it has become possible to produce sugar beet resistant to rhizomania. The public’s concerns about the unexpected effects of transgenic crops have made it a necessity to assess the biosafety of such crops. Also, as a requirement for release certificate, we need to show as many pieces of evidence as possible to justify the substantial equivalence of transgenic events desired to be released.

Having reconfirmed the transgenesis (Fig. 1) that are presumed to activate gene silencing against CP21 of BNYVV in transgenic events, over 320 repeatable spots were detected out of which 32 proteins were found with remarkable alterations in accumulation levels. It should be noted that since sugar beet roots were harvested in the fifth month, when the roots store a lot of sugar, the number of resolved spots were quite low in general.

UniProt data demonstrated that proteins have different biological functions and are a member of diverse metabolic pathways. The main functional group was related to metabolism and defense and stress responses. Neither toxin nor allergen was distinguished except for the spot number 9, which was recognized as phenylcoumaran benzylic ether reductase, Pyrc5. It is related to lignan biosynthesis and plant defense response that leads to protective function against oxidative damage, which may cause allergy in human.^24,25^ Interestingly, it was decreased in the genetically modified S6 plant which could be advantageous for biosafety assessment.

Four proteins including cysteine protease inhibitor 10, hypersensitive-induced response protein 4, lectin, and antiviral protein S are involved in the defense against pathogens, in particular viruses, whose expressions were induced in transgenic plants. Simultaneous accumulations of these proteins during gene silencing against BNYVV are interesting.

The expression of 1-aminocyclopropane-1-carboxylate synthase, catalyzing the conversion of a precursor of ethylene, S-adenosyl-L-methionine (SAM), into 1-aminocyclopropane-1-carboxylate (ACC), was repressed in S3. Accumulation of ACC has been reported to occur during diverse stresses in higher plants.^26^ ACC synthase is encoded by multigene family. Therefore, the other isoenzymes may be active in ethylene synthesis in S3 plants. The expression of pyridoxal 5ʹ-phosphate synthase subunit PDX2, having glutaminase activity, was also repressed in S3. This enzyme catalyzes the synthesis of pyridoxal 5ʹ-phosphate (vitamin B6), a potential oxidant, thereby giving the plant the resistance to singlet oxygen-producing processes.^27^

Tyrosine–tRNA ligase 1 and eukaryotic translation initiation factor 3 subunit B play active roles in protein biosynthesis. Tyrosine–tRNA ligase catalyzes activation tyrosine by ATP to form Tyr-AMP which is then transferred to tRNA (Tyr).^28^ Eukaryotic translation initiation factor 3 subunit B is part of the eIF-3 complex which is active during the initiation stage of translation in eukaryotes.^29^ The former protein expression was seemingly repressed in S6 and increased in S3, while the latter was apparently inhibited in S3. Also, proteasome subunit beta type-2, whose expression was up-regulated in both transgenic events, is one of the important subunits of the proteasome complex that participates in forming a proteolytic environment for protein degradation.^30^

Aspartate aminotransferase P2, soluble inorganic pyrophosphatase, and fructokinase-1 are involved in metabolism. Aspartate aminotransferase P2, not detected in wild type, catalyzes a reversible reaction in which aspartate and 2-oxoglutarate are exchanged for oxaloacetate and glutamate, thus playing an important role in the metabolism of carbon and nitrogen.^31^ Probable fructokinase-1, whose presence was up-regulated in S6, catalyzes the transfer of a phosphate group to fructose and plays a role in continuing the flux of carbon toward starch formation.^32^ Soluble inorganic pyrophosphatase (sPPase) catalyzes the hydrolysis of one pyrophosphate to two phosphate and releases heat. Therefore, it is involved in recycling of the pyrophosphate, a by-product in many cell reactions.^33^ This protein was apparently repressed in S3 and down-regulated in S6.

Four proteins that function in DNA- and RNA-related processes and whose expressions were up-regulated in the transgenic plants include CST complex subunit STN1 which protects the end of chromosomes (telomere) from degradation,^34^ replication factor C subunit 4 as a component of replication factor C (RFC) which causes high-speed DNA synthesis,^35^ ribonuclease J that removes RNAs with inefficient transcription in chloroplasts by exoribonuclease activity. In this sense, it is involved in the development of the chloroplast by regulating gene expression,^36^ and putative pentatricopeptide repeat-containing protein, which is involved in mitochondrial mRNA processing.^37^ These proteins may boost gene silencing process upon activation in transgenic plants.

Proteins associated with cellular transport were identified as follows: Putative aluminum-activated malate transporter 11, Adenine nucleotide transporter BT1, and vacuolar protein sorting-associated protein 22 homolog 1. Putative aluminum-activated malate transporter 11 was absent in S6 event. Most members of aluminum-activated malate transporter family do a range of different functions, but some of them transport malate and inorganic anions from the cytosol to the apoplasm, thereby causing aluminum toxicity tolerance.^38^ Adenine nucleotide transporter BT1, a translocator that exports adenine nucleotides synthesized inside plastids, was induced in transgenic events, while not being present in wild type.^39^ Vacuolar protein sorting-associated protein 22 homolog 1 had different expression levels in transgenic events. Vacuolar protein sorting-associated protein 22 is part of ESCRT-II which is involved in multivesicular bodies (MVBs) formation and endosomal cargo proteins sorting into them.^40^

Numerous studies, both on plants obtained from traditional plant breeding and on crops resulting from genetic engineering, have revealed that no system for genetic modification is without unexpected effects but the notion that any unexpected effect means harmful is a misconception.^41^A review of publications comparing the proteomes of transgenic versus non-transgenic plants showed a range of results. In many studies, the differences were minor,^42–50^ while in some others the changes were substantial.^51–53^ However, in most of these researches, no toxic or allergenic proteins were identified.^43,46,48–50,52,54,55^ These varieties and differences in the results of proteomic evaluations can be due to the type of plant or the purpose of the genomic change^45^ or dependent on the method used to produce genetically modified plants. For example, *Agrobacterium tumefaciens* treatment caused fewer genomic variations than those generated by cell electroporation or particle bombardment.^53^

In addition, previous investigations have reported that such unintentional effects are not limited to genetically modified plants. Such effects related to a single gene expression in transgenic plants are less than differences in plants produced via conventional breeding methods.^43,44^ For example, proteome variations between hybrids and their corresponding inbred lines have been generally observed.^55^ Also, natural genotypic varieties showed much greater differences in gene expression due to simple nucleotide or structural variations.^56^ Furthermore, numerous studies have shown that the effects caused by environmental factors or conditions on protein profile are greater than or similar to the effects of single gene insertion.^45–47,49,52,53^

To know whether the observed changes in protein expression in transgenic sugar beet events compared to the wild type are less than alterations due to environmental factors such as biotic and abiotic stresses, studies on protein profiles of sugar beet under stress conditions was reviewed. For instance, protein extracts of sugar beet genotypes resistant and susceptible to *Fusarium oxysporum* were compared with control samples in two and five days postinoculation by Larson and her colleagues.^58^ Approximately 8% (in susceptible genotype) and 12% (in resistant genotype) of the total proteins detected were induced by fungus. In both genotypes, some proteins had stage-dependent expression patterns as well. BNYVV-induced sugar beet proteins expressions were evaluated by,^4^to understand the interaction between the plant and the disease agent. Using multidimensional liquid chromatography and tandem MALDI-TOF-MS, about 1000 proteins were detected in roots, 11% and 7.4% of which were affected by the virus in susceptible and resistant varieties [R30_Rz1 and R30_rz1], respectively. These proteins were related to defense, oxidative stress response, stress/hormone response, photosynthesis, gene expression, metabolism, signal transduction, and plant development. The aim ofwork was examination of the root tip proteome in response to Fe deficiency. More than 140 spots were detected. Since expression levels of 61 proteins had been altered, iron deficiency caused dramatic changes in the protein profile. For example, dimethyl-8-ribityllumazine (DMRL] synthase which was absent in Fe-sufficient condition, was abundant in root tip under Fe-deficient condition. Yang and his colleagues^57^ analyzed sugar beet monosomic addition Line M14 protein profile in root under salt stress (500 mM NaCl) for one week using 2D-DIGE. 36 protein spots demonstrated considerable changes, of which 12 spots were down-regulated and 24 spots were up-regulated. The proteins were involved in 11 molecular functional groups.

In agreement with the above findings, the low number of altered proteins (less than 8% change) in this study suggests that the unintended effects of an extra gene insertion and activated RNA silencing are minimal and these limited differences could be traced back to expected differences among plant lines (variety-specific differences). These variations fall commonly within the natural range of differences observed in wild plants under diverse environmental conditions and in conventional breeding cultivars.^16^ Hence, the protein composition of genetically engineered sugar beet is substantially equal to the wild type one and our present research was consistent with other investigations. Still, utilizing complementary methods such as metabolomics seems necessary.

## Conclusion

Comparing the protein profiles transgenic plants to their parental wild type plant showed less than 8% changes. None of these recognized proteins were allergens or toxic proteins except for a spot identified Pyrc5, which was decreased in the genetically modified S6 event. These were within the natural range of variations reported in wild plants under diverse environmental conditions and in conventional breeding cultivars.
